# Effect of isavuconazole on the concentration of tacrolimus in a patient with genotype CYP3A5*1/*3: a case report

**DOI:** 10.1186/s40780-025-00427-4

**Published:** 2025-03-13

**Authors:** Hayato Yokota, Yumiko Akamine, Harumi Hatakeyama, Hideaki Kagaya, Sho Sakamoto, Mitsuru Saito, Masahide Takeda, Kazuhiro Sato, Katsutoshi Nakayama, Masafumi Kikuchi

**Affiliations:** 1 Department of Pharmacy, Akita University Hospital, 1-1-1 Hondo, Akita, 010-8543 Japan; 2 Department of Respiratory Medicine, Akita University Graduate School of Medicine, Akita, Japan; 3 Department of Urology, Akita University Graduate School of Medicine, Akita, Japan

**Keywords:** CYP3A5, Drug–drug interactions, Isavuconazole, Tacrolimus, Therapeutic drug monitoring

## Abstract

**Background:**

Azole antifungals are the standard treatment for pulmonary mycosis, which may develop during long-term immunotherapy for kidney transplant. Isavuconazole (ISCZ) is a cytochrome P450 (CYP) 3 A inhibitor that has a risk of interacting with the immunosuppressive drug tacrolimus (TAC). We report a case of simple pulmonary aspergilloma with renal dysfunction due to increased trough levels of TAC after ISCZ coadministration.

**Case presentation:**

A male in his 60s was treated with TAC 3.0 mg/day orally to prevent graft rejection after kidney transplantation. He received a loading dose of ISCZ 600 mg/day orally for two days, followed by a maintenance dose of 200 mg/day for simple pulmonary aspergilloma. The TAC trough concentration increased markedly from 2.4 to 9.9 ng/mL on day 6 after coadministration. The creatinine level increased from 0.70 to 1.08 mg/dL, suggesting renal dysfunction due to TAC. Subsequently, the TAC dosage was reduced, leading to a decreased blood TAC concentration and improved renal function. The patient’s genotype was CYP3A5*1/*3.

**Conclusions:**

In the early stages of ISCZ treatment, the blood TAC concentration is higher, and *CYP3A5* polymorphisms may partially explain the extent of this interaction. We recommend more careful monitoring of TAC and serum creatinine levels for approximately one week after ISCZ administration.

## Background

Tacrolimus (TAC), a calcineurin inhibitor, is an immunosuppressive agent used to prevent graft rejection after kidney transplantation [1]. The therapeutic range of blood TAC concentrations is narrow [2, 3], and long-term exposure to high doses can lead to calcineurin inhibitor-induced nephrotoxicity [4]. Therefore, clinicians individually adjust TAC doses based on therapeutic drug monitoring (TDM) to prevent serious adverse events and graft rejection [5].

Patients who undergo kidney transplantation may develop pulmonary mycosis due to prolonged immunosuppressive therapy [6]. In Europe and the United States (US), the recommended first-line therapy for chronic pulmonary aspergillosis (CPA) is azole antifungals, such as voriconazole (VRCZ) and itraconazole (ITCZ) [7, 8]. In Japan, VRCZ and isavuconazole (ISCZ) are each recommended as first-line therapy [9]. ISCZ is the newest triazole antifungal; it was approved by the US Food and Drug Administration and the European Medicines Agency in 2015 to treat invasive aspergillosis and mucormycosis. ISCZ was approved in Japan in 2023 and is also indicated for the treatment of CPA and cryptococcosis. In a randomized, open-label study comparing ISCZ with VRCZ as a control, the overall response rate at the end of treatment for patients with CPA in the ISCZ group and the VRCZ group was 82.7% and 77.8%, respectively [10]. Drug-related adverse events were reported in 61.5% of the ISCZ group and 85.2% of the VRCZ group. Another retrospective study suggested fewer adverse events with ISCZ than with VRCZ [11]. Therefore, this drug is expected to become a new treatment option for fungal infections, including CPA.

Antifungal agents, such as ISCZ, VRCZ, and ITCZ, are known cytochrome P450 (CYP) 3A4/5 inhibitors that induce interactions with TAC, a substrate of CYP3A4/5 [12]. ISCZ has low CYP3A4 inhibitory activity compared with VRCZ and ITCZ [13]. Although ISCZ demonstrated moderate inhibition when combined with TAC, a 2.3-fold increase in the area under the TAC blood concentration-time curve occurred with ISCZ coadministration in healthy subjects [14], which may indicate the need for TAC dose adjustment. Previous pharmacokinetic studies have shown that the TAC dose must be reduced to maintain the TAC concentration in the therapeutic range with coadministration of ISCZ [15, 16]. However, marked interpatient variability existed in the degree of drug interaction. The concentration of TAC in the blood exhibits considerable variability among patients [17]. The largely inter-individual variability in the pharmacokinetics of TAC can be explained by a single nucleotide polymorphism (SNP) in *CYP3A5* [18]. Zhang Y et al. have shown that in patients who were coadministered VRCZ, the dose-normalized trough concentrations of TAC (C_0_/D ratio) were higher in patients with the CYP3A5*3/*3 allele than in those with the CYP3A5*1 allele [19], whereas ISCZ coadministration has not been investigated. Furthermore, little is known about the variability in TAC concentrations during the coadministration of ISCZ in clinical practice in patients with simple pulmonary aspergilloma.

Here, we report a case of a patient with simple pulmonary aspergilloma who showed an increase in blood TAC levels and renal dysfunction with combined administration of TAC and ISCZ. Furthermore, the patient’s *CYP3A5* polymorphisms were analyzed.

## Case presentation

**Fig. 1:**
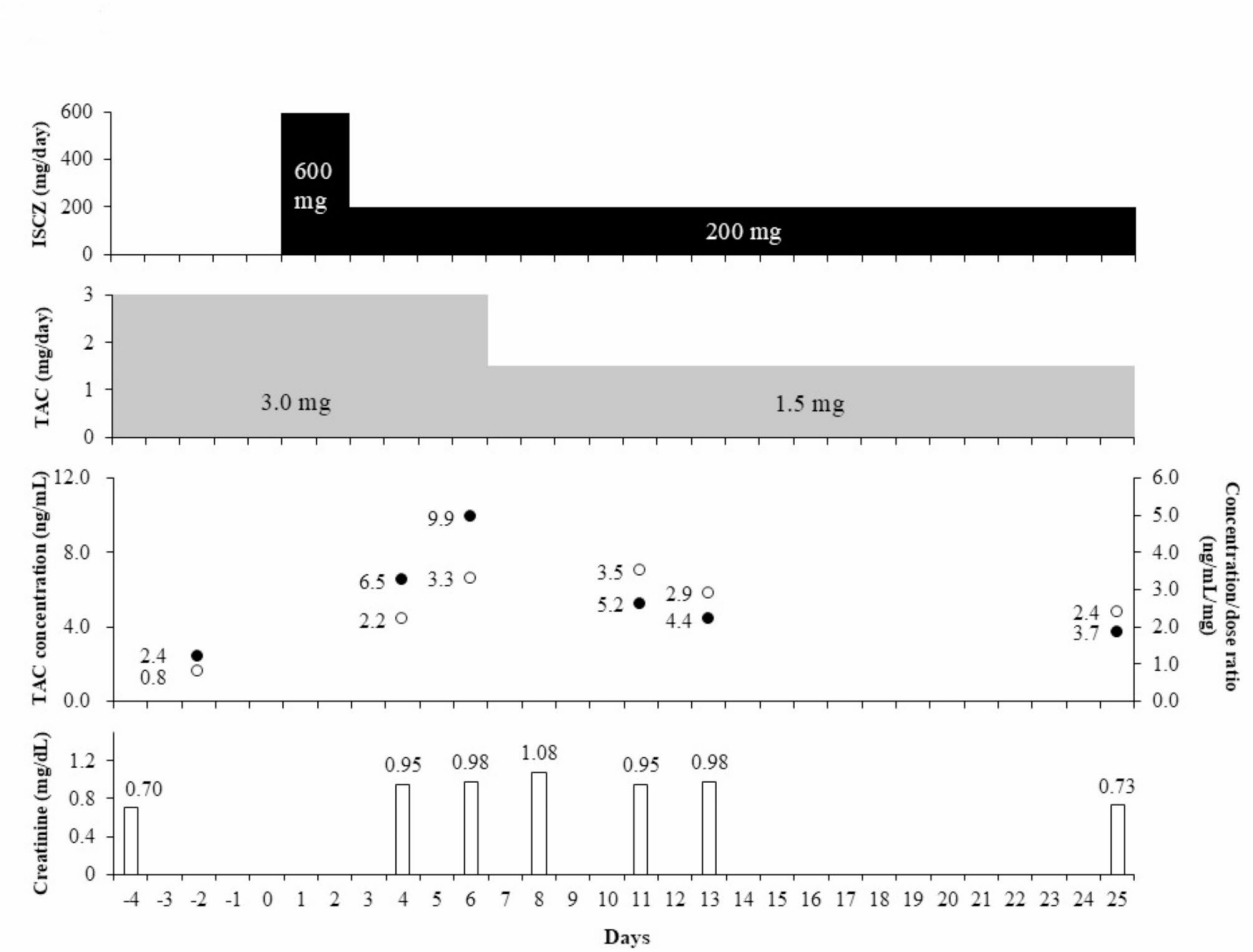
Clinical course of the patient. The doses of isavuconazole (ISCZ) and tacrolimus (TAC) and blood TAC concentrations (black circles), concentration/dose ratio (white circles) and laboratory parameters are shown

A man in his 60s was treated with steroids for nephrotic syndrome with membranoproliferative glomerulonephritis 11 years before the current event. The patient underwent kidney transplantation for end-stage renal disease three years prior to the current event. Immunosuppressive treatment consisted of a regimen of TAC, everolimus, mycophenolate mofetil, and prednisolone. Computed tomography performed during treatment revealed an internal cavity in the right upper lobe of the lung containing a mass with a solid nodule within the pulmonary cavity. The patient underwent a bronchoscopy examination after admission. *A. fumigatus* was detected in the bronchoalveolar lavage fluid, and he was diagnosed with simple pulmonary aspergilloma. After the diagnosis was confirmed, ISCZ was considered as a preoperative treatment. The patient received immunosuppressive maintenance therapy with modified-release formulations of TAC 3 mg/day, mycophenolate mofetil 1000 mg/day, and prednisolone 10 mg/day; everolimus had been discontinued. He also received concomitant bifidobacterium 2 g/day, rabeprazole 10 mg/day, amlodipine 5 mg/day, sitagliptin 50 mg/day, repaglinide 0.5 mg/day, miglitol 100 mg/day, and lemborexant 5 mg/day. Lemborexant was pre-reduced to 2.5 mg/day because of concerns about interaction with ISCZ. All other drugs were continued at the same dose. Before the administration of ISCZ, the trough blood concentration of TAC was 2.4 ng/mL (target trough level: around 5 ng/mL), and the C_0_/D ratio was 0.8 ng/mL/mg, with a creatinine level of 0.70 mg/dL. Oral ISCZ was initiated with a 2-day loading dose of 200 mg three times daily, followed by a daily dose of 200 mg. On day 4 after the start of ISCZ therapy, the trough blood concentration of TAC increased to 6.5 ng/mL (Fig. 1). On day 6, it rose to 9.9 ng/mL (C_0_/D ratio: 3.3), which was well above the target concentration. We suspected TAC-induced nephrotoxicity because the creatinine level rose to 0.98 mg/dL. With blood trough levels and laboratory markers indicative of TAC toxicity, the TAC dose was reduced the next day, from 3.0 to 1.5 mg/day. Liver function tests were within the normal range (aspartate transaminase 20 U/L, alanine transaminase 28 U/L, total bilirubin 0.9 mg/dL). The patient was without clinical symptoms and did not require additional treatment. Serum creatinine levels increased to a maximum of 1.08 mg/dL but subsequently decreased after a slight delay following the reduction in TAC dosage. On day 5 after the TAC dose reduction (in Fig. 1, day 11), the TAC blood level decreased to 5.2 ng/mL (C_0_/D ratio: 3.5), and the patient was discharged on day 13 (C_0_: 4.4 ng/mL, C_0_/D ratio: 2.9 ng/mL). On day 19 after the TAC dose reduction (in Fig. 1, day 25), during an outpatient visit, renal function was observed to have recovered (creatinine: 0.73 mg/dL), and the TAC concentration had returned to therapeutic levels (TAC C_0_: 3.7 ng/mL, C_0_/D ratio: 2.4).

This case was approved by the Ethics Committee of Akita University School of Medicine (no 1015 and 3207). The patient gave his consent for the publication of this report. According to the manufacturer’s instructions, blood TAC concentrations were measured using a chemiluminescence enzyme immunoassay on an Architect-i1000 instrument (Abbott Laboratories, Abbott Japan Co. Ltd.). For genotyping of the *CYP3A5* 6986 A > G (*3) SNP, the polymerase chain reaction-restriction fragment length polymorphism method was used [20]. The patient was determined to have a CYP3A5*1/*3 genotype.

## Discussion and conclusions

Here, we report a case of *simple* pulmonary aspergilloma in which drug-drug interactions between ISCZ and TAC led to increased TAC blood levels, followed by the development of renal dysfunction. This case also focused on *CYP3A5* polymorphisms to explore the drug–drug interaction mechanism. ISCZ is a moderate CYP3A4/3A5 inhibitor [21]. ISCZ has lower CYP3A4 inhibition than VRCZ, ITCZ, and fluconazole in liver microsomes, with an inhibition constant of 0.622 µmol/L [22, 23]. However, the CYP3A4 metabolic pathway for TAC in patients with the CYP3A5*1/*3 genotype, unlike that in patients with the CYP3A5*1/*1 genotype, is expected to be susceptible to CYP3A4/5 inhibition [24, 25]. This suggests that similar results may be observed with the use of ISCZ. Furthermore, in patients with the CYP3A5*3/*3 genotype, the TAC blood concentration increase is higher than that in patients with the CYP3A5*1/*1 and CYP3A5*1/*3 genotypes [19, 24]. The results of this interaction suggest that TAC trough levels are extremely higher with TAC and ISCZ combination treatment in patients with the CYP3A5*3/*3 genotype. Meanwhile, ISCZ is a substrate of CYP3A4 and CYP3A5, with 33.8% and 68.4% residual ISCZ, respectively [21]. Léa Darnaud et al. reported that ISCZ clearance in patients with the CYP3A5*3/*3 genotype is lower than that in patients in the phase 1 and phase 3 SECURE trials [26, 27]. A further study with more focus on *CYP3A5* polymorphism analysis is necessary to quantify the percentage of TAC dose reductions needed when ISCZ is initiated.

Although the trough concentration of TAC before ISCZ administration tended to be lower than the target concentration, the dose of TAC was not increased, due to concerns about an interaction with ISCZ. A previous study showed that the TAC C_0_/D ratio increased 1.44 times on the second day after ISCZ administration [28]. Therefore, in this case, we considered it unlikely that TAC dose adjustments would be necessary within the first two days after ISCZ administration. ISCZ requires a two-day loading dose, and the TAC C_0_/D ratio reaches a maximum on the fourth day of coadministration [15]. Thus, we needed to monitor the TAC blood concentration carefully, which was first confirmed on the fourth day of coadministration. In this case, on day 6, the blood TAC concentration and C_0_/D ratio increased approximately fourfold from baseline. In a study of patients who had undergone allogeneic hematopoietic stem cell transplantation, the C_0_/D ratio of TAC increased within 7 days of ISCZ coadministration, and no significant differences compared to baseline were observed after the second week [29]. In a study of solid organ transplant recipients, in the early stages of ISCZ treatment, a decrease in TAC dosage occurred in 61.3% of patients, but after that, TAC levels remained stable from baseline to one month [30]. Therefore, management of varying blood TAC concentrations is critical for approximately one week after the initiation of coadministration of TAC and ISCZ.

On day four following the initiation of ISCZ treatment, a TAC concentration-dependent increase in creatinine levels was observed, requiring a reduction of the TAC dose by half on day 7. An objective causality assessment based on the Horn Drug Interaction Probability Scale revealed a probable interaction between TAC and ISCZ [31]. High TAC intra-patient variation is associated with a noticeable decrease in renal function and affects CD4+/CD8 + cells, indicators of immune status [32]. Acute kidney injury during hospitalization increases the risk of rehospitalization and increases the risk of overall mortality [33]. Moreover, it has been reported that the TAC maximum trough level was associated with acute kidney injury during admission. Therefore, during TAC administration, we should minimize concentration fluctuations by monitoring TAC trough values to prevent worsening renal function.

According to FDA drug labeling, when VRCZ is used concomitantly with TAC, the TAC dose should be reduced to one-third or lower [34]. In contrast, there is no recommended empirical dose reduction for TAC when coadministered with ISCZ. Fernández-Ruiz M et al. and Monforte A et al. have reported that the daily TAC dose was reduced by 30–50% when ISCZ was coadministered early in treatment [30, 35]. Other studies have suggested that a reduction in the TAC dose of 18% may be recommended [28]. However, the range of TAC dose reductions considered in these reports varies widely, and the reports are not in agreement. Therefore, reducing the TAC dose prophylactically by a defined amount is inappropriate. In the present case, we did not reduce the TAC dose before the start of ISCZ coadministration, but frequent TDM after ISCZ administration may have allowed the patient to continue treatment without discontinuing TAC. Hiratsuka et al. reported that the genotype frequencies of CYP3A5*1/*1, *1/*3, and *3/*3 in Japanese individuals are 7.9, 35.5, and 55.9%, respectively [36]. The allele frequency could support the importance of frequent monitoring of TAC concentrations. Furthermore, *CYP3A5* polymorphism analysis may help clinicians improve their understanding of the severity of these interactions. ISCZ is often preferred over other azole antifungals in terms of interactions and is expected to gain more clinical experience in the future.

When initiating administration of a combination of ISCZ and TAC, we recommend that the blood TAC concentration and creatinine levels be carefully monitored for approximately one week. This case shows that the severity of drug interactions may partially be explained by *CYP3A5* polymorphism.

## Data Availability

The datasets used and/or analysed during the current study are available from the corresponding author on reasonable request.

## Abbreviations

C_0_/D: dose-normalized trough concentration
CPA: chronic pulmonary aspergillosis
CYP: cytochrome P450
ISCZ: isavuconazole
ITCZ: itraconazole
TAC: tacrolimus
TDM: therapeutic drug monitoring
US: United States
VRCZ: voriconazole
